# Is “Congestion of the Brain” a Correct Pathological Expression?

**Published:** 1882-03

**Authors:** 


### Is "Congestion of the Brain" a Correct Pathological Expression?

In the course of his Croonian lectures on the influence of the circulation on the nervous system, delivered last spring before the Royal College of Physicians of London, Dr. Walter Moxon answers the question very emphatically in the negative. After a very instructive analysis of the various anatomical, physical, and physiological elements involved in the subject, Dr. Moxon continues as follows:

"Can you in any individual case prove by post-mortem appearances that death was caused by congestion of the brain, or even that a state of congestion of the brain or over-fullness of its blood vessels preceded death? I believe these questions must be answered firmly in the negative. In establishing such a negative, one has to meet with a very strong prejudice, rooted in most tenacious grounds—the grounds of convenience and of ancient and universal acceptance. After examining a body dead from brain symptoms, when all you are able to see in your examination is only that the veins are very full of blood, it is very convenient to be able to say that death was caused by congestion of the brain, and it sounds much better for a skilled witness than to say that death was caused by insensibility; so that the doctrine of congestion of the brain is convenient, and the universality of its acceptance may be illustrated by the naive earnestness with which authors of great works adopt it. . . . We find writers giving four conditions as showing congestion. The first is the swollen state of the brain, so that it seems after removal from the calvaria almost too large for the cavity which contained it. The dura mater seems tightly stretched over it, and, on reflecting this, the convolutions appear broad and flattened, and the sulci less obvious. No allusion is made here to hypertrophy of the brain, which is known only, so authors say, by its causing a general enlargement of the whole organ. I have never seen such hypertrophy, but the flattening described has very frequently come under my observation, but always in the presence of some obvious cause of expansion of the brain, in the form, usually, of an increase of the intra-ventricular fluid, or else of apoplectic bleeding or of tumor, in which case the swelling and flattening are more localized.

"The next evidence (of congestion of the brain) mentioned by writers is the distention of the veins and capillaries with blood, the veins are tortuos and varicose, the gray matter dark. Both this and the white matter show abundance of bloody points and gorged vessels, and the description ends with, 'It is extremely difficult to draw the line and say what is morbid and what is consistent with health.' Now, it is better, nay, it is necessary, to say firmly that it is simply impossible to draw the line and to say what is consistent with health. But the question is not about the degree of health to be inferred from a post-mortem, but whether the mode of dying, the position of the body after death, or the manner of making the inspection, will not determine the appearance of extreme overfullness of the vessels of the brain of the dead person, independently of the conditions which were antecedent to all this. Can we infer from the amount of blood in the brain after death the amount during life? Kussmaul and Tenner, after numerous experiments, under various conditions and circumstances accurately predetermined, declare as the result of their investigations that they could not deduce any results from post-mortem examinations undertaken to ascertain the state of fullness before death of the most important parts of the vascular system. Now, if this is the conclusion where the conditions before and after death were determined and known, how can it be said that the amount of blood found after death in the human brain will show the amount present before death, when the conditions before and after death were unknown and undetermined?"

The author applies the same line of reasoning to other organs —to the liver and to the stomach, with special reference to the appearance caused by sudden death by heart disease. He then continues: "During life, redness, when associated with swelling, heat, and pain, are at one with these associates in proving the existence of inflammation; but after death the redness, which was due to the enlarged scope of vascular play, ceases, because vascular play has ceased with life. The consequence is not doubtful, and it is quite certain that after death the redness goes from inflamed and congested parts. Such serious issues may turn upon this point that I think it very necessary to clearly recognize that no degree of redness, or pinkness, or over-fullness of blood about the brain or its membranes, can prove that there was any morbid state of the circulation within the head before the act of dying. Death by asphyxia increases the amount of blood in the head, but a dependent position of the head after death, if only for a short time, will cause a similar increase. It should be taught that it is a sign of ignorance to say in a coroner's court that congestion of the brain was found on post-mortem examination to be the cause of the person's death."

These emphatic words have an obvious medico-legal application. To those who are in the habit of making autopsies with what is deemed proper thoroughness for judicial purposes, and who have invariably opened the head of the cadaver with that end in view, and have noted and described, with more or less faithful attention to details, the blood supply of the brain and its meninges as an essential part of the examination, these new teachings will suggest the propriety of revising the usual interpretation of injected blood vessels. But the observations of Moxon cannot serve to justify the examiner in omitting to examine the contents of the cranium in every instance of post-mortem inquiry in death by violence, no matter how evident the cause of death may seem to have been declared upon inspection of other regions of the body. If the medical jurist desires an excuse for saving himself extra labor with the saw, mallet and knife, when he thinks he has found the cause of death in some region below the head, he will find a distinguished American precedent in the record of the autopsy of the late President Garfield; it will be remembered that before proceeding to the inspection of the body in that case it was "unanimously agreed not to open the head."—*Boston Med. and Surgical Journal.*

